# Learning to Predict Perceptual Distributions of Haptic Adjectives

**DOI:** 10.3389/fnbot.2019.00116

**Published:** 2020-02-06

**Authors:** Benjamin A. Richardson, Katherine J. Kuchenbecker

**Affiliations:** Haptic Intelligence Department, Max Planck Institute for Intelligent Systems, Stuttgart, Germany

**Keywords:** haptic intelligence, perception, ordinal regression, tactile sensing, predicting probability distributions, haptic adjectives

## Abstract

When humans touch an object with their fingertips, they can immediately describe its tactile properties using haptic adjectives, such as hardness and roughness; however, human perception is subjective and noisy, with significant variation across individuals and interactions. Recent research has worked to provide robots with similar haptic intelligence but was focused on identifying binary haptic adjectives, ignoring both attribute intensity and perceptual variability. Combining ordinal haptic adjective labels gathered from human subjects for a set of 60 objects with features automatically extracted from raw multi-modal tactile data collected by a robot repeatedly touching the same objects, we designed a machine-learning method that incorporates partial knowledge of the distribution of object labels into training; then, from a single interaction, it predicts a probability distribution over the set of ordinal labels. In addition to analyzing the collected labels (10 basic haptic adjectives) and demonstrating the quality of our method's predictions, we hold out specific features to determine the influence of individual sensor modalities on the predictive performance for each adjective. Our results demonstrate the feasibility of modeling both the intensity and the variation of haptic perception, two crucial yet previously neglected components of human haptic perception.

## 1. Introduction

Much of modern machine learning focuses on modeling tasks for which inputs are sorted into discrete categories, such as image classification for visual data and speech recognition for audio data (e.g., Deng et al., 2009; Goodfellow et al., 2016). In the domain of haptics, machine learning is mainly used to pursue similar classification tasks in which models aim to recognize specific objects or surfaces from tactile data (Fishel and Loeb, 2012; Spiers et al., 2016). Typically, a model is trained on a large amount of raw tactile data that are manually labeled; given new tactile data, it can then predict the object or surface from which the data were captured. Although haptic recognition is an important task that humans perform well (Klatzky et al., 1985), it is limited in its applications because the classification categories are constrained to a specific set, which restricts the experiences that can be recognized and prevents generalization. For example, if a robot is trained to recognize specific textures or objects, it has no way to identify anything that it hasn't experienced before.

Given the limitations of recognition tasks, learning higher level semantic attributes will likely benefit generalization; these attributes could include structural haptic cues, like size, or substance-related adjectives, like hardness and texture (Klatzky et al., 1987). Because they are more discriminable dimensions than structural cues in a purely haptic setting (Klatzky et al., 1987), this work focuses on substance-related haptic adjectives.

A number of haptics researchers have used machine learning to try to teach robots to identify *haptic adjectives* from raw, multi-modal tactile sensor data (Chu et al., 2013, 2015; Bhattacharjee et al., 2018). In each of these cases, objects are labeled by humans with various binary haptic adjectives like hard or rough, and raw data are gathered when a tactile-sensor-equipped robot interacts with the objects. Then, machine learning is used to train models to map measurable *features* (characteristics) of the tactile data to the human *labels*. The methods used in all these cases have at least the following three drawbacks:

1. The features that are calculated or extracted from the raw data are carefully hand crafted. Their design often requires expertise, and they are developed for specific tasks, which limits how well they generalize to other tasks.
2. The binary labels (e.g., hard or not hard) are determined either by thresholding measured mechanical properties such as stiffness (Bhattacharjee et al., 2018) or by taking the consensus of binary labels provided by multiple humans (Chu et al., 2013, 2015). In either case, a rich, continuous perceptual space for humans is reduced to a much simpler binary space for an artificial system, which requires selection of an arbitrary threshold and ignores any perceived differences in the strength of attributes.
3. Associating a single label with a trial ignores the natural variability in perception across individuals and interactions. A self-aware human recognizes that some other people would respond differently and might even be able to estimate the distribution of reactions a population would provide.

In reference to the first drawback, various methods exist for extracting representations from raw data without relying on carefully designed features. Neural networks can extract many levels of abstracted representations from data while making very few assumptions about the underlying structure (Goodfellow et al., 2016). However, the learned representations typically depend to some extent on the specific training task. While research in transfer learning has shown that learned representations can be transferable to other tasks (Pan and Yang, 2010; Bengio, 2012), other methods can find underlying structure independently of any task. Autoencoders, for example, learn representations of data by compressing raw data into a lower-dimensional space and then uncompressing the middle layer to match the input data as closely as possible (Hinton, 2006). Variational autoencoders (VAEs) work similarly, but they represent data points as parameterized probability distributions over a latent space (Kingma and Welling, 2014). Another type of feature-extracting algorithm, unsupervised dictionary learning, has been successfully used to extract features from raw tactile data for multiple haptic classification tasks (Madry et al., 2014). We additionally demonstrated the viability of these methods in our previous work (Richardson and Kuchenbecker, 2019), in which the learned features greatly outperformed hand-crafted features in the binary adjective classification tasks presented by Chu et al. (2015). We use the same unsupervised dictionary feature-extraction algorithms in this work. While we acknowledge that other unsupervised learning methods, such as autoencoders or VAEs, could discover equally or more powerful representations of data, that is not the focus of this paper.

Regarding the second drawback, a standard way to capture richer information about human perception is to allow human raters to classify samples with discretization levels that are finer than a binary decision. One experimental method that yields this richer information is a sorting task. By allowing raters to sort materials by similarity and then analyzing the results using multidimensional scaling, Bergmann Tiest and Kappers (2006) were able to compare perceived compressibility and roughness across many different materials. Hollins et al. (1993) used a similar procedure to determine that hardness/softness and roughness/smoothness are primary, orthogonal dimensions of tactile perception, and that springiness, or the elasticity of a material, might correspond to an additional primary dimension. Using similar methodology, Hollins et al. (2000) identified sticky/slippery as a third, less salient dimension of tactile perception. Another method is to have subjects rate tactile stimuli on a scale. Motivated by the lack of consensus regarding the antonymous relationship between haptic adjectives (e.g., hard vs. soft) and the primary dimensions of tactile perception (Picard et al., 2003; Guest et al., 2010), Chu et al. (2015) had subjects rate 60 objects on a five-point rating scale for 10 distinct adjectives. They chose 10 adjectives that have been considered by various researchers to represent relevant perceptual dimensions, but they never analyzed or published these results. This paper will summarize the experiment used to gather the data, analyze these ordinal labels, and use machine learning to learn and predict them.

In reference to the third drawback, there are a variety of ordinal regression and classification algorithms that attempt to model the latent variable underlying the ordinal data (Gutiérrez et al., 2016). However, these approaches typically account for a variable that underlies the entire distribution of responses. In the case of the labels gathered by Chu et al. (2015), each of the 60 objects has its own distribution of labels for each attribute, which depends on both the object and on the entire underlying perceptual distribution of that attribute. Said another way, different people have different opinions about how to apply specific descriptions. For example, some people might say a particular blanket is soft, while others perceive it to be very soft. With enough data, these variations across people can be captured. Thus, given a single interaction with an object, it should be possible to predict the distribution of labels that interaction and object would receive if experienced by a large number of people. Such functionality would be useful for companies selling tangible products to quickly understand how a particular material will be perceived by a range of possible customers. However, we could not find any algorithm that can predict a distribution of responses from a single interaction; all of them predict single labels.

**Figure 1:**
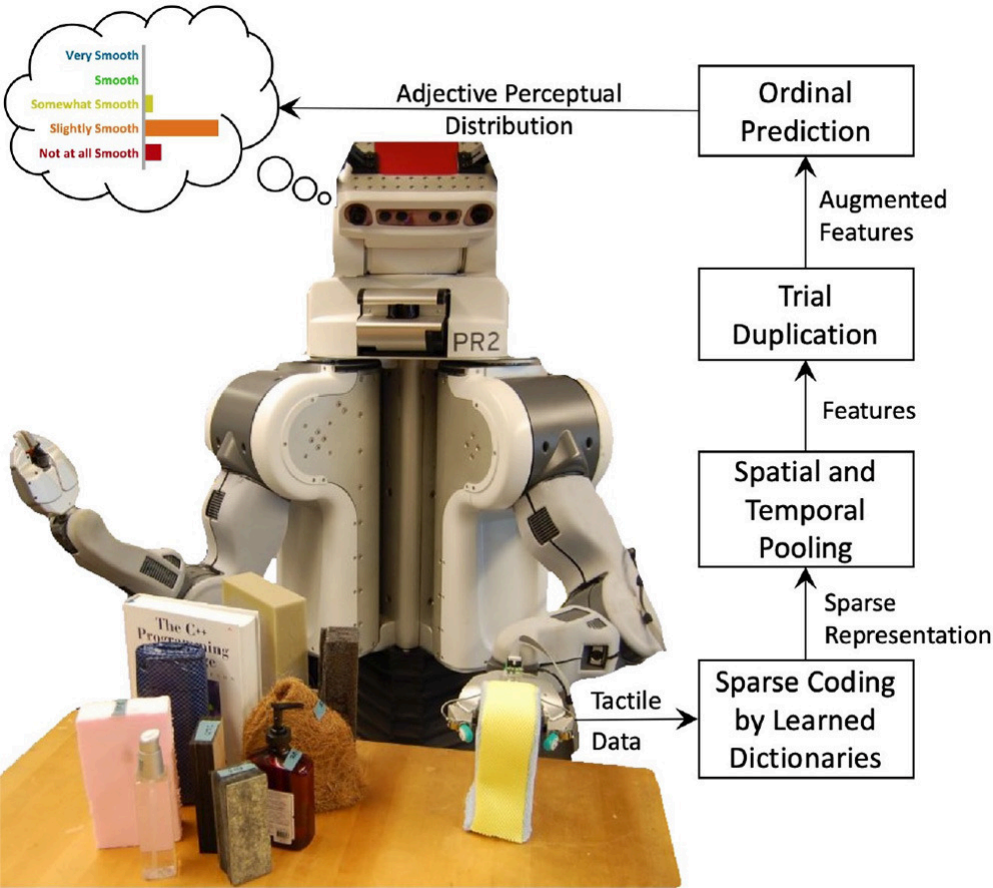
Predicting the perceptual distribution of a haptic adjective. How **smooth** will this object feel to a range of people? Features are extracted from the robot's raw tactile data. These are augmented to include a bias parameter ranging from 0 to 1. Finally, ordinal regression predicts a probability density over the five adjective ratings.

The main goal of this paper is to train models that accurately map tactile data to distributions of ordinal haptic adjective labels. We use unsupervised dictionary-learning methods to extract representative features from raw tactile data, and we develop a modified ordinal regression method to model the relationship between the features and label distributions. A general overview of the prediction process is shown in Figure 1. Following our previous work (Richardson and Kuchenbecker, 2019), we measure the contribution of different exploratory actions and haptic sensor modalities to the learning and prediction of the adjectives. The secondary goal is to analyze the labels gathered by Chu et al. (2015) and provide insight into the antonymous relationships between common haptic adjectives.

## 2. Materials and Methods

Throughout this paper, we rely on a number of algorithms and newly designed methods to process and model the rich haptic data in the Penn Haptic Adjective Corpus-2 (PHAC-2) dataset (Chu et al., 2015). Section 2.1 describes the experimental procedure that was used to gather the data, as well as the methods we used to analyze the labels. As explained in section 2.2, dictionary-learning algorithms are used to extract features from raw tactile data because they have proven effective for representing tactile data for a range of tasks. Section 2.3 proposes a new method to incorporate object-specific ordinal label distributions into model training. Finally, section 2.3.3 describes how the method is used in an existing ordinal regression framework.

### 2.1. The PHAC-2 Dataset

In an effort to understand the relationship between raw tactile information and human perception of haptic interactions with objects, Chu et al. (2015) collected the PHAC-2 dataset using two similar experiments. For the first, a robot equipped with state-of-the-art tactile sensors repeatedly touched 60 objects. For the second, human subjects explored the same 60 objects in controlled conditions, providing multiple types of haptic descriptions for each object. The experiments were designed to provide the robot and humans with maximally similar experiences.

The 60 objects were selected from everyday items and constructed from common materials with the goal of providing a wide range of tactile experiences that would stay consistent throughout the study. To be included, an object had to be able to stand stably on a table and provide two approximately parallel, vertical, opposing surfaces with the same uniform texture. All objects are between 1.5 and 8.0 cm thick and at least 10 cm tall to facilitate two-fingered exploration. The selected objects can be clustered into the following eight categories: 16 foam objects, 5 organic objects, 7 fabric objects, 13 plastic objects, 12 paper objects, 2 stone objects, 2 glass objects, and 3 metal objects.

Although Chu et al. (2015) fully described the human-subject experiment, they did not discuss or publish all of the results. Because we present some of these unpublished results, we will provide a summary of the robot experiment followed by a full description of the human-subject experiment.

#### 2.1.1. Robot Exploration

**Figure 2:**
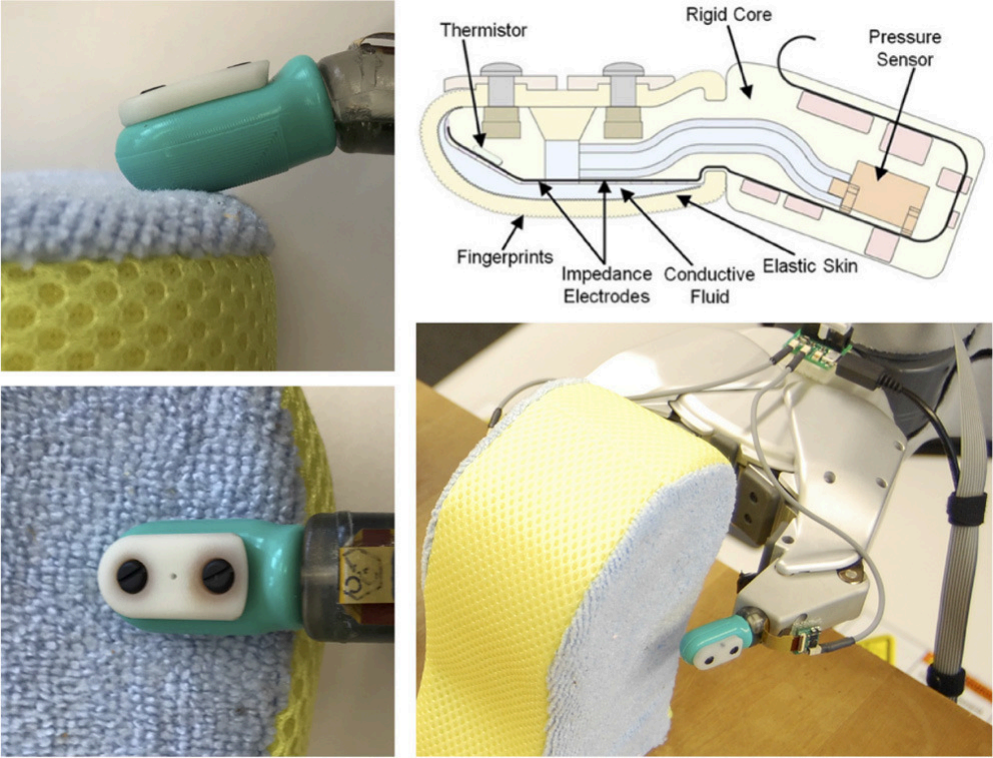
Detailed views of the BioTac-equipped PR2 hand interacting with the Blue Sponge object, and a diagram showing the internal components of the BioTac sensor.

As shown in Figure 2, a Willow Garage Personal Robot 2 (PR2) equipped with two BioTac tactile finger sensors (SynTouch LLC) was used to gather multi-modal haptic data. It performed an identical series of interactions with each of the 60 objects 10 times, for a total of 600 trials. The BioTac, which is designed to imitate the sensing capabilities of a human fingertip, measures overall pressure, vibration, temperature, heat flow, and fingertip deflection. The robot performed the same four exploratory procedures (EPs) (Lederman and Klatzky, 1993) for each trial in the following order: *Squeeze, Hold, Slow Slide*, and *Fast Slide*. These EPs were designed to imitate the frequently used human EPs of Pressure, Static Contact, and two speeds of Lateral Motion. Because humans prefer to determine distinct object properties using individual EPs (Lederman and Klatzky, 1993), it is reasonable to expect that certain robot EPs might discriminate some object properties better than others. Each BioTac measured the absolute steady-state fluid pressure (*P*_*DC*_), dynamic fluid pressure (*P*_*AC*_), steady-state temperature (*T*_*DC*_), heat flow (*T*_*AC*_), and voltages on 19 spatially distributed impedance-measuring electrodes (*E*_1:19_). *P*_*AC*_ was sampled at 2.2 kHz, and the other channels were sampled at 100 Hz. To perform *Squeeze*, the PR2 slowly closed its gripper at constant velocity until the value of *P*_*DC*_ reached a predefined threshold, after which it slowly opened the gripper to the original position. During the *Hold* EP, the gripper was closed for 10 s to a position that was halfway between the gripper distance at initial contact with the object and at the *P*_*DC*_ threshold during *Squeeze*. To perform *Slow Slide* and *Fast Slide*, the gripper was closed by 20 and 10%, respectively, of the *Squeeze* distance, moved downward by 5 cm at 1 and 2.5 cm/s, respectively, and then released. A video of the robot exploring the Satin Pillowcase object can be found in the Supplementary Materials. For a more detailed description of the robot experiment, please see Chu et al. (2013, 2015).

#### 2.1.2. Human-Subject Study

To capture how humans describe haptic interactions, thirty-six people took part in an experiment in which they haptically explored objects and provided descriptions. All procedures were approved by the University of Pennsylvania's Institutional Review Board under protocol #816464. Subjects gave informed consent and were compensated $15 for participation. The cohort of participants contained 34 right-handed and 2 left-handed people, with 10 males and 26 females between the ages of 18 and 21 years. All subjects were students at the University of Pennsylvania and had normally functioning arms and hands.

##### 2.1.2.1. Experimental procedure

**Figure 3:**
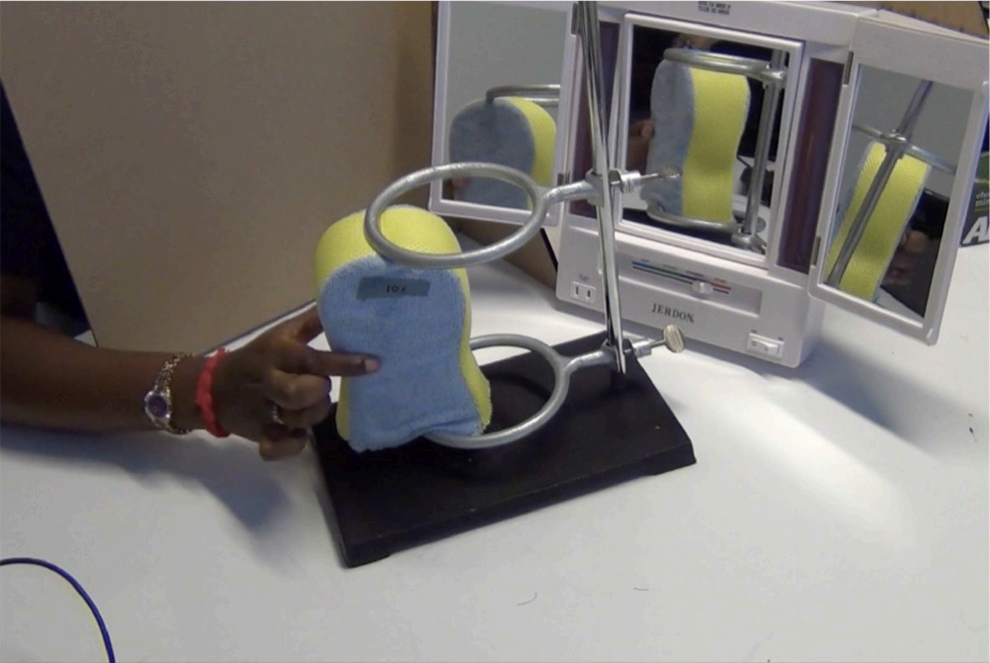
A human subject touching the Blue Sponge object during the experiment.

The subject sat at a table at which the objects were presented. Individual objects were suspended from a ring stand above the table surface so that the subject could neither lift nor move the object. A large vertical panel prevented the subject from seeing their hand or the object. Additionally, the subject wore noise-canceling headphones playing white noise to block ambient noise and any sound generated during interaction with the objects. To imitate the limitations of the PR2, the subject was instructed to use only their thumb and index finger from one hand. Additionally, they were allowed to use only a fixed set of exploratory procedures when probing the objects: pressure, enclosure, static contact, and lateral movement. Figure 3 shows an image of a subject mid-experiment. A video of this subject-object interaction can be found in the Supplementary Materials. Because Chu et al. (2015) wanted to understand natural perceptually grounded language, subjects were not coached in any way about how to define or apply the haptic adjectives used in the study.

To make the experiments more manageable, the 36 subjects were split into three groups of 12, each of which was assigned a unique set of 20 objects (one third of the full set of 60 objects). The 12 participants from each group interacted only with the 20 objects assigned to their group. For each participant, the experiment was split into two stages. The first was used to familiarize the subject with the procedure, and the second was used to gather concrete data. In both cases, all 20 objects were presented in a random order, and the subject touched a compliant stress ball between each object to cleanse his or her haptic “palate.” In the first stage, the subject freely described the feeling of each object to the experimenter. In the second stage, the subject was asked to rate each object on both binary and scaled ratings of pre-determined haptic adjectives while they were interacting with the object. The subject first selected the binary labels from a list of 25 haptic adjectives that were displayed in random order on a screen. Then the subject rated the object on a five-point scale for the 10 basic haptic adjectives **hard**, **soft**, **rough**, **smooth**, **slippery**, **sticky**, **cold**, **warm**, **moldable**, and **springy**. These scaled ratings were collected to test whether certain basic haptic adjectives have antonymous relationships and can be considered to lie along relevant tactile dimensions. The 25 binary haptic adjectives were investigated in detail by Chu et al. (2015); however, the scaled ratings were not studied. In this work, we will present and discuss the scaled adjective ratings for the first time.

##### 2.1.2.2. Scaled adjective ratings

**Figure 4:**
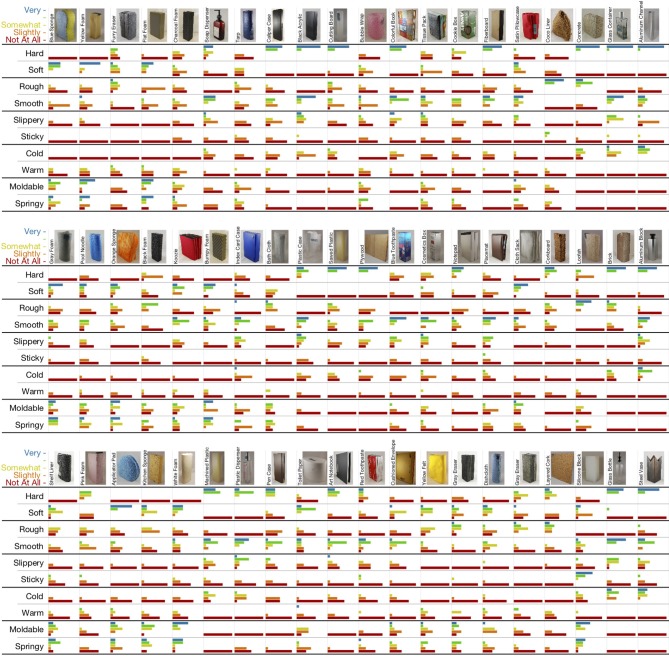
The 60 objects of the PHAC-2 dataset along with all the scaled adjective ratings given by subjects. The objects are shown in the same three groups of 20 that were used in the study. Colored bar length is proportional to the number of responses the indicated rating received. At a glance, it is clear that **hard** and **soft** are antonyms, whereas **moldable** and **springy** seem to be synonymous.

Each of the 60 objects was rated on a scale that included 1 – “not at all (e.g., hard)”, 2 – “slightly (hard)”, 3 – “somewhat (hard)”, 4 – “(hard)”, and 5 – “very (hard)”, for the 10 basic haptic adjectives listed above. These adjectives are considered by some to comprise five basic antonym pairs that lie along relevant, and in some cases primary, dimensions of tactile perception (Hollins et al., 2000; Okamoto et al., 2013). The posited antonym pairs are **hard** – **soft**, **rough** – **smooth**, **slippery** – **sticky**, **cold** – **warm**, and **moldable** – **springy**. The full set of responses for all 60 objects is shown in Figure 4, including the names and small pictures of the objects.

In this paper, we will analyze the collected haptic adjective ratings on their own, and then we will deeply explore whether features extracted from the robot's raw tactile data can be used to learn distributions over scaled adjective ratings. When considering the ratings alone, we first wanted to investigate how well subjects agreed on how to apply each set of scaled haptic adjective ratings to each object. We quantified interrater agreement for each adjective-object combination by calculating *r*_*wg*_, the most common such metric used in the literature (O'Neill, 2017). It is defined as:

(1) $$\begin{array}{r} r_{wg}=1-\frac{S_{X}^{2}}{\sigma_{eu}^{2}}=1-\frac{S_{X}^{2}}{\left(\frac{A^{2}-1}{12}\right)}, \end{array}$$

where *S*_*X*_ is the observed variance in the subjects' ratings with the chosen adjective scale on the chosen object and σ_*eu*_ is the variance of the null distribution, which we set to the variance of a uniform distribution across our *A* = 5 categories. This metric is equal to one when all subjects choose the same adjective rating for an object, and it is zero when they choose randomly among the categories. Negative values indicate less agreement than what stems from random guessing; we do not set negative values to zero, as is sometimes done, to preserve the information provided by the calculation (O'Neill, 2017).

Second, given the uncertainty in the current literature, we investigated the extent to which subjects actually used the five adjective pairs as antonyms; we were particularly uncertain about the antonym relationships between **slippery** and **sticky**, and between **moldable** and **springy**, which have not been firmly established as antonym pairs. We investigated this question by calculating Spearman's rank-order correlation, ρ, between all possible pairs of adjective ratings. Spearman's ρ is a nonparametric measure of rank correlation, similar to the Pearson product-moment correlation for parametric data; we calculated it using the MATLAB function corr with the “type” option set to “spearman.” The magnitude of the resulting value shows the strength of the association between the two involved adjectives, with values near zero indicating no correlation. The sign of ρ shows the direction of the association; synonyms have a large positive correlation, while antonyms have a large negative correlation. We also evaluate the *p*-value associated with each observed correlation, using α = 0.05 to determine significance.

### 2.2. Unsupervised Feature Learning

**Figure 5:**
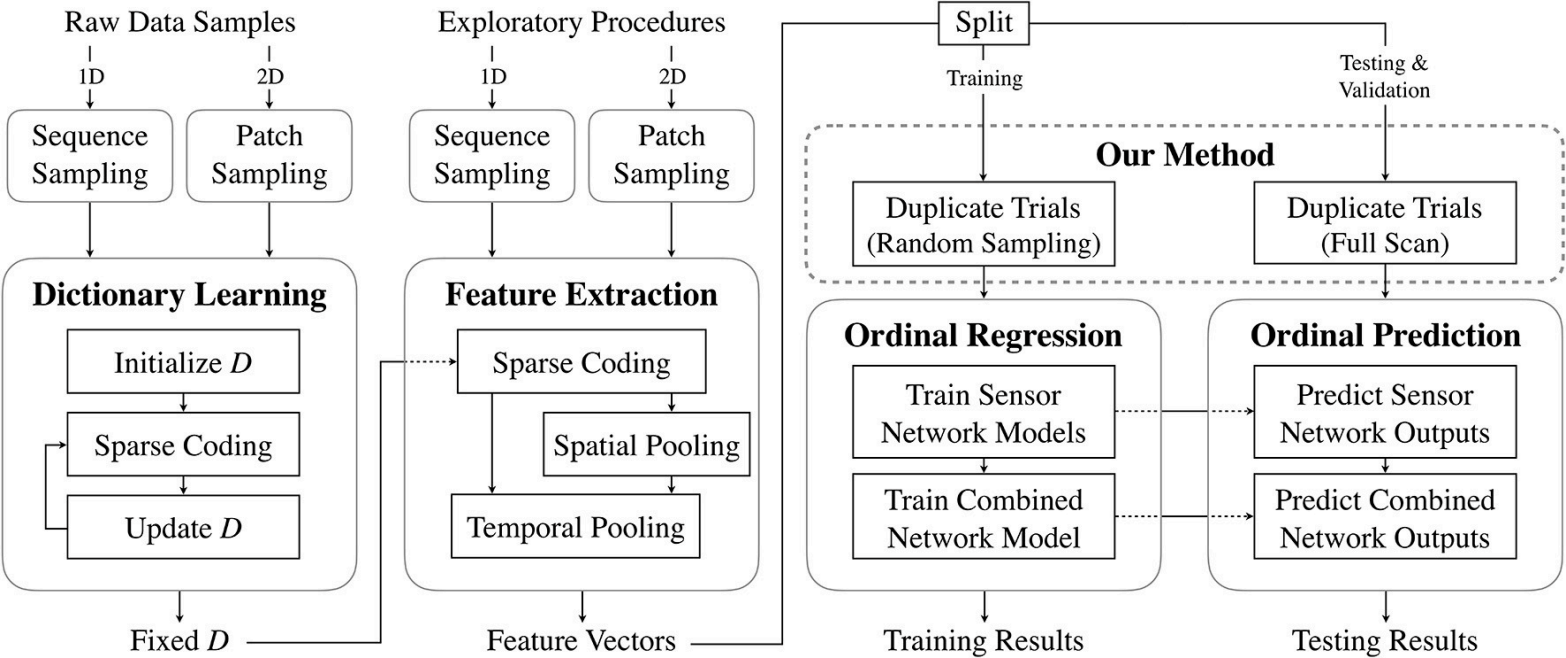
Summary of the data-processing pipeline. Samples from raw sensor data (either subsequences or patches of 1D and 2D signals, respectively) are collected and used to learn dictionaries in an unsupervised manner. These are then used to extract features from full exploratory procedure trials. A subset of the feature vectors is used to train neural networks to perform ordinal regression. The learned models are tested on a distinct subset of feature vectors.

To map the robot sensor data to the human adjective ratings, we first need to distill relevant information, or features, from the raw data. The full learning process from raw data to prediction of label distributions is shown in Figure 5. Although our primary contribution pertains to the method mapping the learned features to the labels, this section describes the process used to extract the features from the raw data, shown in the first two columns of Figure 5. Specifically, we used unsupervised dictionary learning, which has proven far more effective than using hand-crafted features (Richardson and Kuchenbecker, 2019).

#### 2.2.1. Description of Dictionary Learning

To learn powerful representations of the raw data, we used the dictionary-learning method K-SVD (Aharon et al., 2006). The goal of K-SVD is to first learn a dictionary composed of unit vectors, called atoms, and then to use the learned dictionary to represent new data as sparse linear combinations of the atoms. More precisely, given a data array $Y=\left[y_{1},\ldots ,y_{M}\right]\in {\text{IR}}^{n\times M}$ with *M* observations of length *n*, K-SVD learns a *K*-atom dictionary $D=\left[d_{1},\ldots ,d_{K}\right]\in {\text{IR}}^{n\times K}$ and the corresponding sparse code matrix $X=\left[x_{1},\ldots ,x_{M}\right]\in {\text{IR}}^{K\times M}$ by solving the optimization problem:

(2) $$\begin{array}{r} \underset{D,X}{\min}∥Y-DX{∥}_{F}^{2}\text{subject to}∥x_{m}{∥}_{0}\leq T,\\ \text{for}m=1,\ldots ,M, \end{array}$$

where ||·||_*F*_ denotes the Frobenius norm, ||·||_0_ denotes the *ℓ*^0^ norm (which counts the nonzero entries), and *T* is the sparsity constraint, which places an upper-bound on the number of nonzero entries in each column of *X*. Given a learned dictionary, K-SVD can compute sparse code matrices for new observations. These matrices can in turn be used as features or pooled to create more abstract features. A high-level overview of the K-SVD process is shown in the first column of Figure 5.

#### 2.2.2. Feature Extraction Procedure

**Figure 6:**
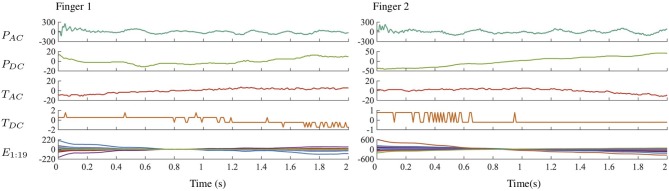
Scalar and electrode signals from the robot's two fingers over time during execution of the *Fast Slide* EP on the Blue Sponge object.

The PHAC-2 dataset contains sequences of four types of scalar data (*P*_*AC*_, *P*_*DC*_, *T*_*AC*_, *T*_*DC*_) and one type of spatially distributed data (*E*_1:19_), all captured during four different EP interactions; a sample recording is shown in Figure 6. Because the dataset contains both scalar and spatial temporal data, two methods were needed to extract features. K-SVD with temporal max pooling was used to extract features from the scalar signals, and Spatio-Temporal Hierarchical Matching Pursuit (ST-HMP) (Madry et al., 2014), an extension of K-SVD, was used to extract features from the spatially arranged electrode signals. After dictionaries were learned on a subset of all the tactile sequences, they were then used to compute sparse code representations of tactile sequences taken from individual trials. We learned one dictionary for each combination of the five sensory data streams and the four EPs, giving 20 total dictionaries. Six randomly selected trials per object, or 60% of the total number of trials, were used to train the dictionaries. Each dictionary was trained on data from only a single sensor signal and a single EP. In total, there are 600 feature vectors for each sensor-EP pair.

To train dictionaries on the scalar signals using K-SVD, the tactile sequences are cut into smaller overlapping vectors of length *n*, each of which is used as a single observation *y*_*i*_ in *Y*. After the learned dictionary is used to extract a sparse codes matrix for an individual tactile sequence, the sparse codes are max pooled over subsequences, or temporal cells, of multiple lengths. Finally, the pooled codes from each cell are aggregated into a single feature vector representing the tactile sequence.

On the other hand, ST-HMP extends K-SVD by performing dictionary learning on frames from temporal sequences of spatially distributed tactile data (Madry et al., 2014). Each frame can be treated as a 2D tactile image, which is partitioned into small overlapping 2D spatial patches. K-SVD is then used to compute an underlying representation of these patches. In the K-SVD framework, the tactile data contained within each 2D patch from every image are treated as a single observation *y*_*i*_ of *Y*. Thus, several columns of Y correspond to a single tactile image. After it is learned, a dictionary can be used to compute sparse code matrices representing the patches. By spatially and temporally pooling the max codes, ST-HMP constructs a feature vector from a sequence of tactile images.

In our specific K-SVD implementation, the values of *n* were chosen to be 22 for the 2.2 kHz *P*_*AC*_ signal and 50 for the 100 Hz *P*_*DC*_, *T*_*AC*_, and *T*_*DC*_ signals. In each case, vectors overlapped by 50% of their length. The dictionary sizes *K* were chosen to be 40 for *P*_*AC*_, 25 for *P*_*DC*_ and *T*_*AC*_, and 10 for *T*_*DC*_. For temporal pooling, we partitioned most of the tactile sequences into 16, 8, 4, 2, and 1 temporal cells for a total of 31 cells. *Fast Slide* sequences of *P*_*DC*_, *T*_*AC*_, and *T*_*DC*_ were not long enough to split into 16 cells, so they were split into only 8, 4, 2, and 1 cells. Because *P*_*AC*_ is sampled at a higher frequency than the other signals, the pooling leads to greater downsampling.

For our implementation of ST-HMP, the 19 BioTac electrode values were arranged into a 7 × 3 array that maintained relative measurement positions; the two additional values needed to complete the matrix were interpolated from the nearby readings, as done by Chebotar et al. (2016). The two resulting 7 × 3 arrays, one from each BioTac finger, were concatenated along one of the long edges to form a 7 × 6 array. ST-HMP was applied to the sequences of these 7 × 6 tactile images. These images were partitioned into 3 × 3 patches, with a scanning step size of 1. The dictionary size *K* was chosen to be 10. For spatial pooling, each tactile image was divided into 9, 4, and 1 cells for a total of 14 spatial cells. The tactile sequences were divided into 16, 8, 4, 2, and 1 cells for a total of 31 temporal cells.

The specific parameter values for K-SVD and ST-HMP were selected because they previously showed good performance in a binary adjective classification task using the same raw tactile data (Richardson and Kuchenbecker, 2019). Additionally, because unsupervised feature learning is not the primary focus of this work, full parameter optimization was not a priority. The full feature extraction procedure, including the sparse coding and the spatial and temporal pooling is summarized in the second column of Figure 5.

### 2.3. Prediction of Perceptual Distributions

To map the extracted features to the distributions of labels, we designed a new method within an ordinal regression framework that can associate information about full label distributions with individual interactions. We can use the learned models to predict a distribution of labels from data gathered during a single interaction. An overview of the full process is shown in the last two columns of Figure 5.

#### 2.3.1. Method: Capturing Perceptual Distributions

As described above, each of the 60 objects has ~12 rated responses for each of the 10 adjectives. With each response selected from five possible rating classes for each adjective, each object can be given a distinct five-dimensional label *L*_*a, o*_ = {*n*_1_, *n*_2_, *n*_3_, *n*_4_, *n*_5_} for each adjective *a*, where *o* represents the object and *n*_*x*_*i*__ is the number of times that the particular rating *x*_*i*_ was chosen by the participants for the selected adjective-object pair.

Given the collected ratings, there exists a unique probability distribution of ratings for any given adjective-object pair, where for a given rating *x*, the $P\left(x|a,o\right)=\frac{n_{x}}{\underset{i}{\sum }n_{x_{i}}}$. Additionally, because the ratings are ordinal, there is a corresponding cumulative distribution function (CDF) defined for a discrete random variable *X* such that $F_{X,\left(a,o\right)}\left(x\right)=P\left(X\leq x\right)=\underset{x_{i}\leq x}{\sum }P\left(x_{i}|a,o\right)$. More generally, the probability of a particular response is a function of the random variable.

**Figure 7:**
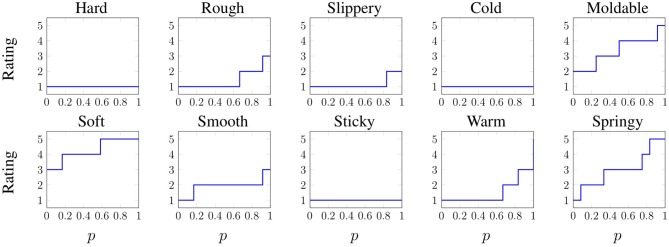
Inverse cumulative distribution function of each adjective for the Blue Sponge object. Recall the meaning of the ratings: 1 – “not at all (e.g., hard)”, 2 – “slightly (hard)”, 3 – “somewhat (hard)”, 4 – “(hard)”, and 5 – “very (hard).”

In order to predict a probability distribution of adjective responses for a single trial, we designed a method that trains a model to learn an approximation of the *inverse* of *F*_*a, o*_(*x*) for all (*a, o*) pairs, along with how that inverse function depends on the features extracted from raw data. Then, given new features, the model can predict the inverse of *F*(*x*) for that specific trial, and thus an approximate distribution of expected responses. The inverse of *F*(*x*) is called the quantile or inverse cumulative distribution function and is defined as *F*^−1^(*p*) = *inf*{*x* ∈ IR:*p* ≤ *F*(*x*)}, *p* ∈ [0, 1]. The inverse CDF for each adjective of the Blue Sponge object is shown in Figure 7. This approach differs from traditional cumulative link models (Agresti, 2002) because it learns an inverse cumulative distribution function for each specific object instead of for an entire population. The method is slightly different for training and validation/testing, and it works as follows.

During model training, each trial feature vector *f*_*t*_ is duplicated a fixed number of times *W*. For each duplicate *f*_*t, w*_, one extra feature $p_{w}\sim U\left\{0,1\right\}$ is added to the end of the feature vector. Thus, each duplicate of a trial is identical except for the last feature. The single labels *x*_*i*, (*t, w*)_ for the modified duplicates are assigned using $F_{o}^{-1}\left(p_{w}\right)$, where *F*_*o*_(*x*) is the cumulative distribution function for the object being explored during that particular trial. One can think of *p*_*w*_ as indicating the position of the rater in the population; it shows in a continuous way whether the associated rating is near the low end, the middle, or the high end of the distribution of all ratings for this interaction.

To predict the distribution of labels for a new trial during testing or validation, the feature vector is again duplicated. However, in this case the extra variable is simply incremented from 0 to 1. For each modified duplicate, one rating is predicted. Therefore, any changes in the predicted rating across duplicates depend only on the added variable. This method can thus predict the inverse cumulative distribution function for single trials. The separate training and testing processes are highlighted in the last two columns of Figure 5.

#### 2.3.2. Ordinal Classification Algorithm

Using the features extracted by dictionary learning and our method for capturing perceptual distributions, we trained models to learn how to predict label distributions for new interactions. Because the adjective ratings are ordinal (i.e., they have a relative order but no defined scale), we use ordinal regression instead of traditional multi-class classification. Ordinal regression accounts for the ordered nature of the ratings, whereas multi-class classification ignores it.

Specifically, we used the proportional odds model neural network (NNPOM) algorithm (Gutiérrez et al., 2014). NNPOM is an extension of the proportional odds model (POM). POM estimates the inverse CDF of ordinal labels as a linear model of the inputs (McCullagh, 1980). NNPOM uses a single hidden layer of neurons between the input and the POM; it thus estimates the inverse CDF as a linear model of nonlinear basis functions from the hidden neurons. We chose this algorithm because it has sufficiently high performance with low training times compared to other common ordinal classification algorithms like support vector machine (SVM) methods.

#### 2.3.3. Implementation Details

With 20 separate feature sets for each combination of sensor modality and EP, it was natural to train an adjective-specific model for each feature set to determine which combinations perform well for which adjectives. We used NNPOM with a sigmoid activation function to train each model. A total of 20 models, one for each feature set, was trained per adjective.

**Figure 8:**
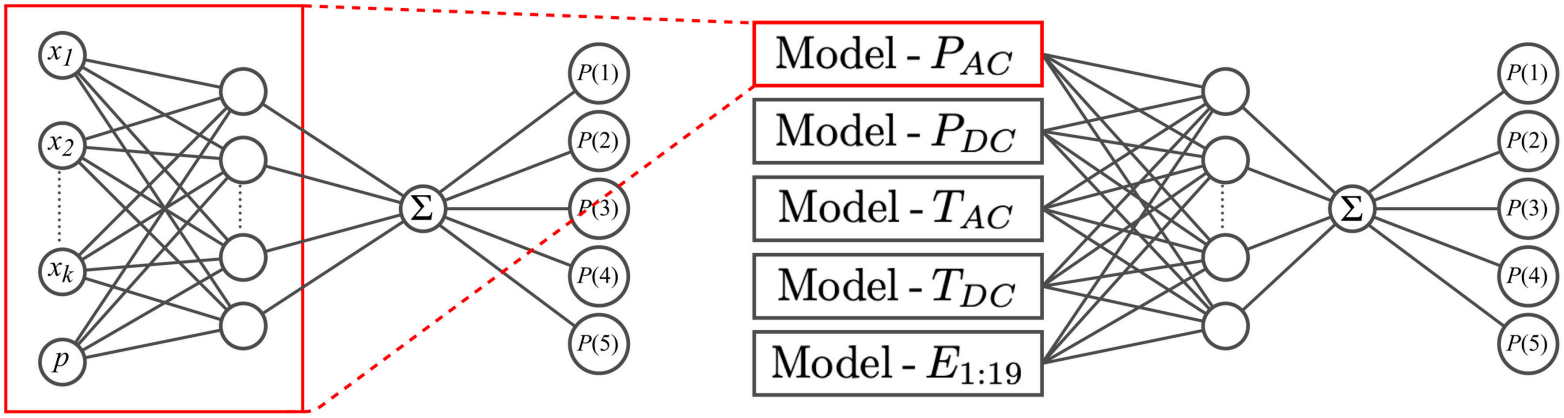
Neural network structure for the individual sensor model and the combined model, each with one hidden layer. The combined model takes as input the outputs from the hidden layers of five individual models, one from each sensor modality.

Because humans perceive tactile interactions as simultaneous combinations of multiple sensation types, it is interesting to determine how each robot sensor modality contributes to the learning and prediction of different haptic properties. For example, do vibration sensors play a more important role than temperature sensors in the perception of **rough** and **smooth**? To learn the contribution of each sensor modality to adjective perception and to determine whether performance is improved by including all sensor modalities in one model, we trained additional NNPOM models for each EP; these models merge one EP's five learned representations from the sensor-specific models. Specifically, the outputs of the hidden layer neurons from the optimized sensor-specific models were used as the inputs to a combined NNPOM model. The structure of the combined model is shown in Figure 8. A total of four fully combined models, one for each EP, was trained for each adjective to measure the overall performance change. To compare the individual contributions of the sensor types, additional combined models were trained while holding out the features from a single sensor (by setting their features all to zero). Five of these holdout models were trained for each EP-adjective pair. To train both the sensor-specific and combined models, we used the NNPOM implementation developed by Gutiérrez et al. (2016).

To train and validate the models, we split the 60 objects into separate training, validation, and testing sets for each adjective. Six objects were used for each of the validation and testing sets, and the remaining 48 objects comprised the corresponding training set. To prevent the classifiers from learning to understand objects instead of adjectives, all 10 trials for each object were kept together in the same set.

We performed cross-validation by training models on the training set and measuring their accuracy on the validation sets. This approach was used to optimize the model parameter *N*, the number of neurons in the hidden layer, over the set {1, 5, 10, 20, 30}, and the parameter λ, the regularization parameter, over the set {0.001, 0.01, 0.1, 1, 10}. During model training the validation error was measured every 10 iterations. After 150 iterations with no decrease in error, the training stopped, and the model with the best performance was kept.

Each model was trained according to the process described in section 2.3.1. Each of the training feature vectors was duplicated 15 times, a different random number p~U{0,1} was added to each duplicate, and the duplicates were labeled using $F_{o}^{-1}\left(p\right)$ of the corresponding object *o* (for a total of 15 duplicates × 10 trials = 150 training examples per object). The validation and test trials were duplicated 99 times with the added extra variable *p* incremented by 0.01 from 0 to 1 noninclusive, and the ground-truth labels were assigned in the same way as they were for the training samples.

Then for each adjective, four EP-specific combined models were optimized, where each model is trained using information from all five sensory modalities. As shown in Figure 8, the outputs of the hidden layers of the optimized sensor-specific models are used as inputs to the combined model. Again, cross-validation was used to optimize *N*, λ, and the number of training iterations. The training, validation, and test sets were again prepared according to the process in section 2.3.1 with some minor changes. In this case, the training trials for the combined model are each comprised of the feature vectors from all five sensor modalities. Each combined trial was duplicated 15 times, and a different random number p~U{0,1} was added to each combined duplicate and copied to each sensor-specific feature vector. The labels for the combined duplicates were assigned in the same way as above, and the validation and testing trials were prepared similarly.

For each of the 40 combined models (4 EPs × 10 adjectives), five additional holdout models were trained to measure the contribution of each sensor modality to the system's overall performance. Each holdout model has the same parameters (*N* and λ) as the corresponding combined model, and the number of training iterations was optimized on the validation set as described above. For each of the five holdout models, the features from a different single sensor model were held out of training and testing. By measuring the difference in test error between the combined model and each of the holdout models, we can measure the relative contribution of each sensor modality. There are a total of 200 holdout models (5 sensor types × 4 EPs × 10 adjectives) in addition to the 40 combined models. For each EP-adjective pair, there are a total of six types of grouped models: the combined (nothing held out), *P*_*AC*_-holdout, *P*_*DC*_-holdout, *T*_*AC*_-holdout, *T*_*DC*_-holdout, and *E*_1:19_-holdout models.

In all validation and testing, the performance of the models was measured by taking the average across all trials of the per-trial macroaveraged mean absolute error (*MAE*^*M*^) metric, as defined by Baccianella et al. (2009). We use *MAE*^*M*^ because it measures error for imbalanced ordinal datasets more precisely than traditional error metrics such as Mean Absolute Error. Specifically, it normalizes the contribution to the error by class. To define it for a single trial *t*, let the set of duplicate feature vectors *f*_*t, w*_ and associated labels *y*_*t, w*_ be denoted *Td*_*t*_, and let *X*_*t*_ be the set of ratings *x*_*i*_ that are represented in *Td*_*t*_. With these definitions in mind, the per-trial *MAE*^*M*^ can be defined as:

(3) $$\begin{array}{r} MAE^{M}\left(\hat{\Phi },Td_{t}\right)=\frac{1}{\left|X_{t}\right|}\underset{x_{i}\in X_{t}}{\sum }\frac{1}{\left|Td_{t,x_{i}}\right|}\underset{f_{t,w}\in Td_{t,x_{i}}}{\sum }\left|\hat{\Phi }\left(f_{t,w}\right)-y_{t,w}\right| \end{array}$$

where $\hat{\Phi }$ represents the learned model, *Td*_*t*,_*x*__*i*__ denotes the set of duplicates with true labels *y*_*t, w*_ = *x*_*i*_, and |*X*_*t*_| and |*Td*_*t*,_*x*__*i*__| denote the cardinality of the respective sets.

## 3. Results

We analyze how the study participants used the scaled haptic adjective ratings, and then we investigate the extent to which features automatically extracted from the raw tactile data can be used to learn distributions over scaled adjective ratings.

### 3.1. Human Perception

**Figure 9:**
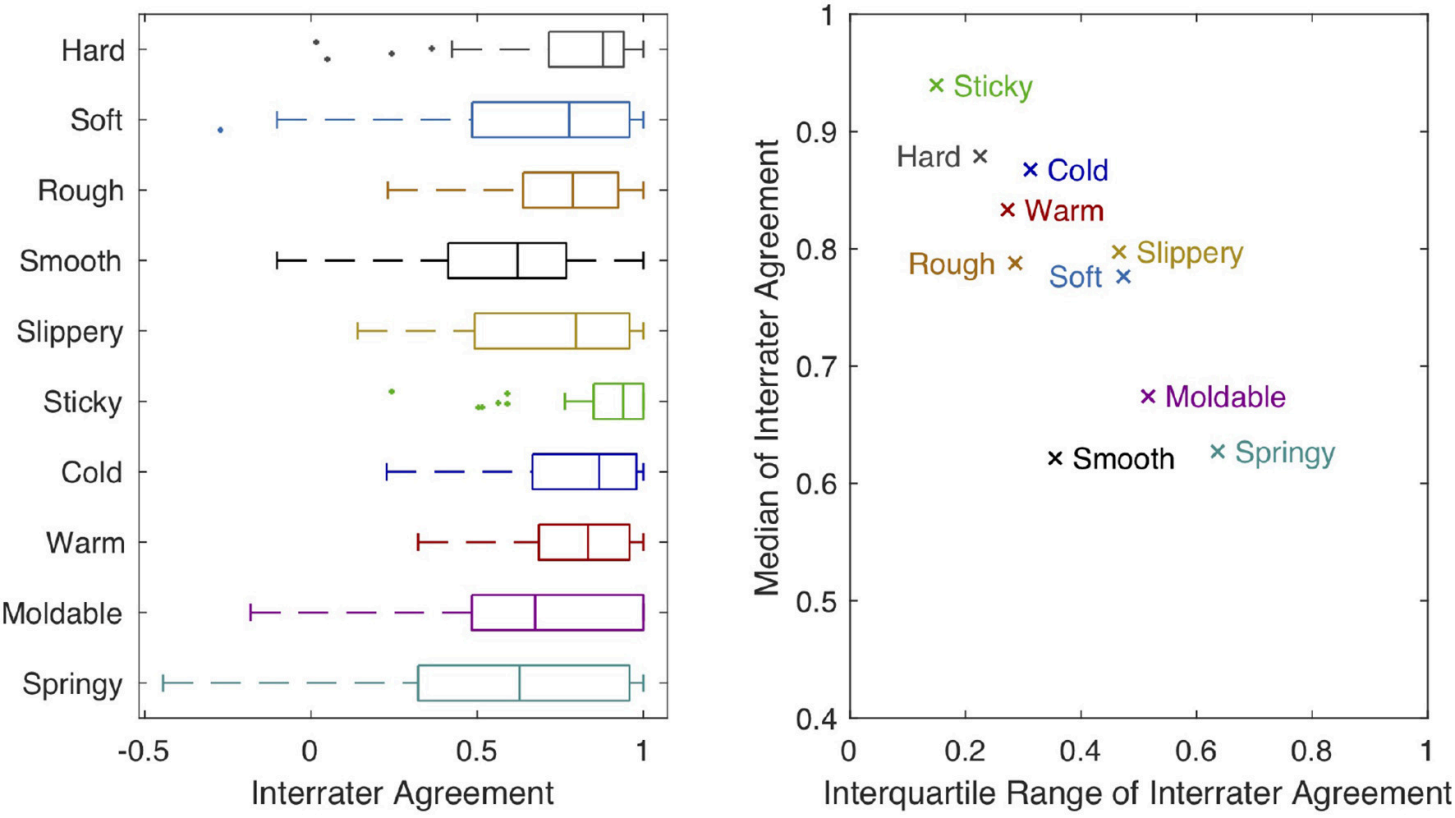
The boxplot on the left shows interrater agreement (*r*_*wg*_) for each of our 10 haptic adjective scales. The central mark of each box indicates the median of the distribution across objects. The edges of the box are the 25th and 75th percentiles; the whiskers extend to the most extreme datapoints that are <1.5 times the interquartile range (IQR) away from the closer 25th or 75th percentile mark. Outlier points outside this range are plotted individually. The graph on the right plots the median of *r*_*wg*_ against its IQR for each haptic adjective scale.

Figure 9 shows the distribution of interrater agreement *r*_*wg*_ across all 60 objects for each of our 10 adjective scales. The adjectives **sticky**, **hard**, **cold**, **warm**, and **rough** all have relatively high median values (> 0.75) and relatively small IQRs (< 0.35). **Soft** and **slippery** also have relatively high medians but more variation across objects. **Smooth**, **moldable**, and **springy** have the lowest medians (< 0.70) paired with higher IQRs.

**Figure 10:**
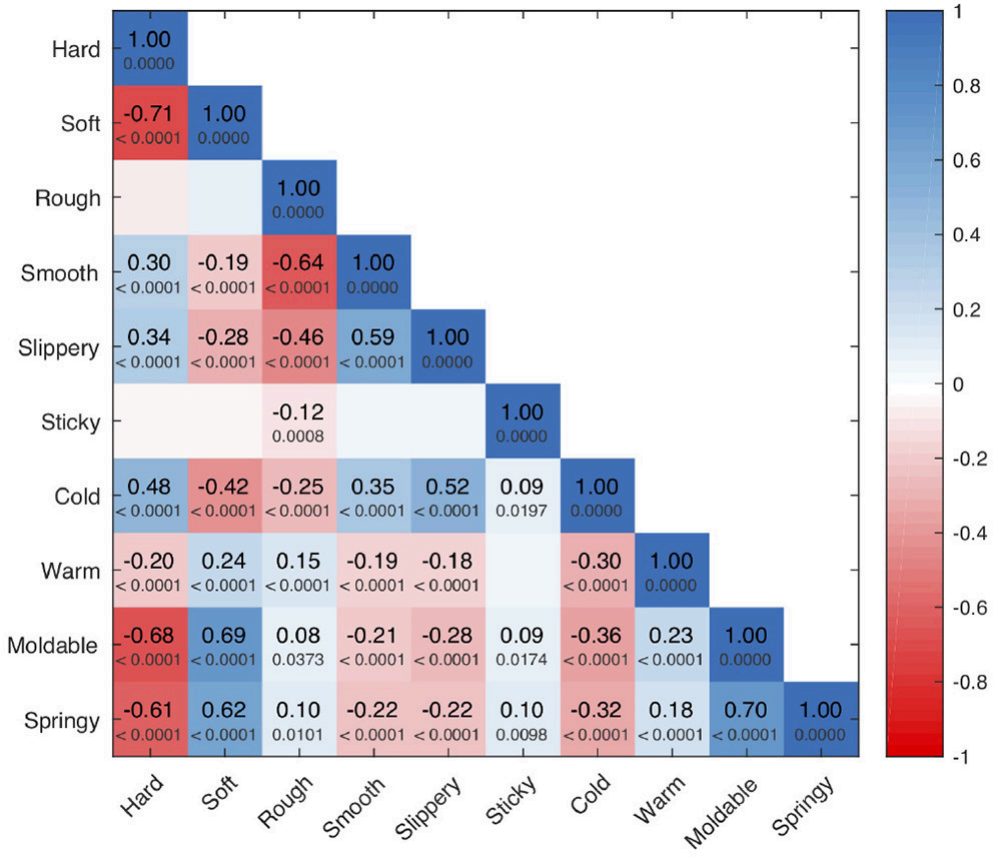
Spearman's rank-order correlation ρ for all pairs of haptic adjective scales, along with the associated *p*-value. To improve readability, we omit these values for insignificant correlations. Boxes showing strong synonyms are colored blue (including the self-synonyms along the diagonal), while strong antonyms are colored red.

Our correlation analysis appears in Figure 10. We see a strong, significant antonym relationship between **hard** and **soft** (ρ = −0.71, *p* < 0.0001), as well as between **rough** and **smooth** (ρ = −0.64, *p* < 0.0001). **Sticky** and **slippery** are uncorrelated. **Cold** and **warm** appear to be weak, significant antonyms (ρ = −0.30, *p* < 0.0001), whereas **moldable** and **springy** show a strong, significant synonym relationship (ρ = 0.70, *p* < 0.0001). Both **moldable** and **springy** are strongly positively correlated with **soft**, showing subjects used these three adjectives largely synonymously. **Slippery** is strongly correlated with **smooth** (and anti-correlated with **rough**), showing that subjects used this pair largely synonymously. **Hard** and **cold** are also significantly positively correlated with **smooth** and **slippery**. Interestingly, **sticky** has no strong positive or negative correlations.

### 3.2. Robot Perception

**Figure 11:**
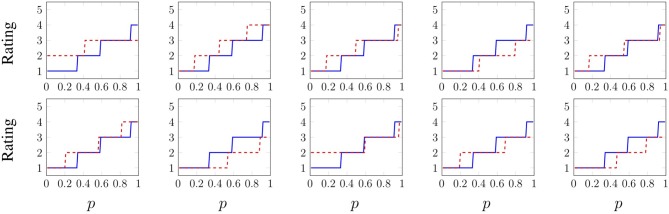
Predicted distributions of the adjective **cold** for all 10 *Fast Slide* trials from the plastic Cutting Board object. The predicted inverse CDFs are shown in dashed red, whereas $F_{\text{cold},\text{CB}}^{-1}$ is shown in blue (and is the same across all 10 trials). The ratings mean 1 – “not at all cold”, 2 – “slightly cold”, 3 – “somewhat cold”, 4 – “cold”, and 5 – “very cold.” The average *MAE*^*M*^ across all 10 trials is 0.4355.

To obtain the following results, models were first trained and optimized on separate training and validation sets. To account for the variation in neural network performance caused by the random initialization of the weights, 10 final models were trained for each of the six types of grouped models (all sensory data streams together plus five holdouts) for every EP-adjective pair, and these models were all evaluated on a testing set that was completely held out during training and optimization. As a sample test-set result from a single combined model, the predicted inverse CDFs of the adjective **cold** for all 10 *Fast Slide* trials from the plastic Cutting Board (CB) object are shown in Figure 11 and compared to $F_{\text{cold},CB}^{-1}$. The average *MAE*^*M*^ across these 10 trials is 0.4355, which is less than half a point on the scale from 1 to 5. Each trial has a different distribution because the recorded tactile data are unique, due to slightly different initial conditions. Some predictions are clearly quite close to the true labels, and in other trials the predicted distribution differs from the true distribution by approximately one rating point.

**Figure 12:**
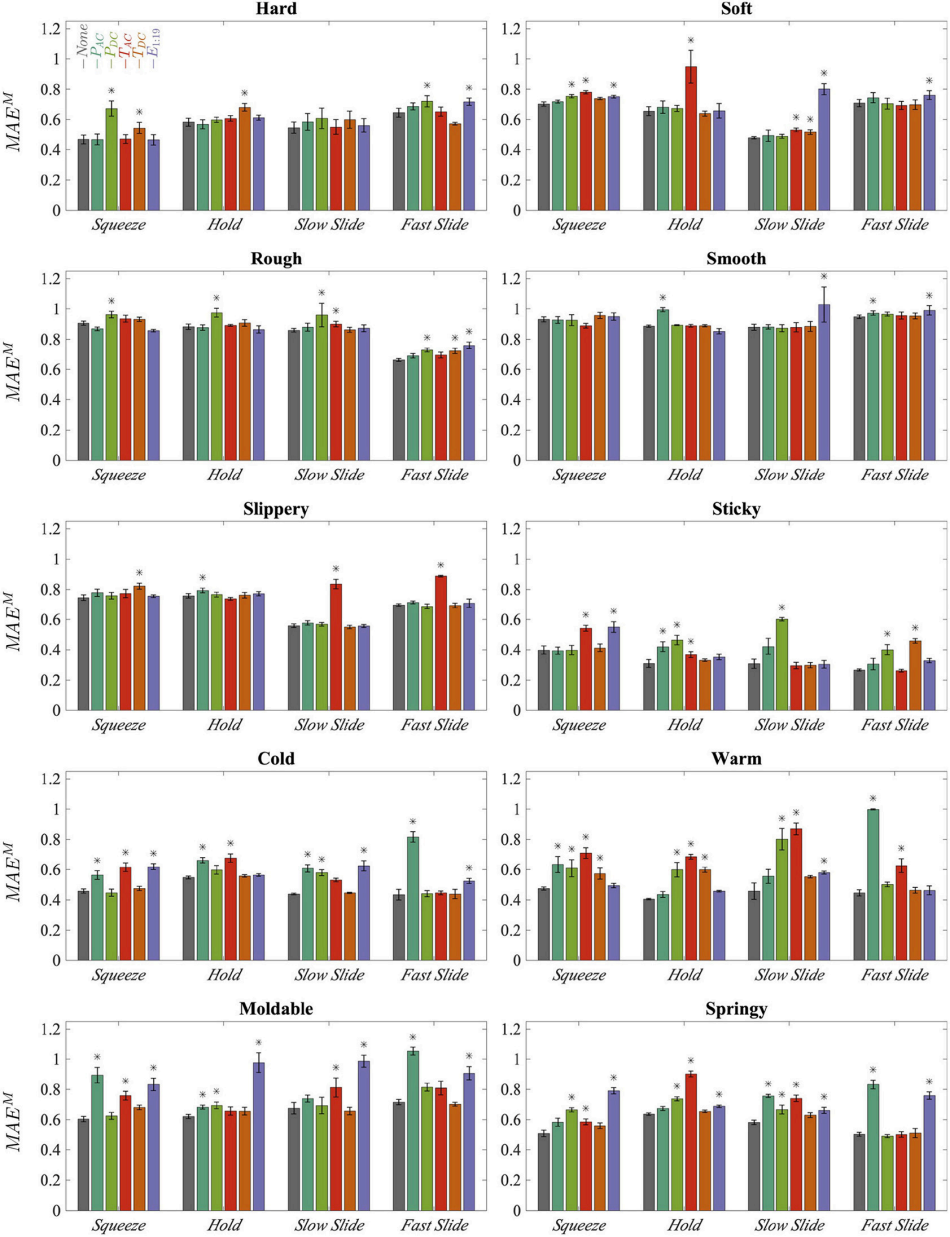
Average error of all 10 trained models for each type of grouped model, sorted by exploratory procedure and adjective; lower error is better. Error bars display standard deviations across 10 trained models. The label of a single bar indicates the sensor type that was held out during model training. Asterisks mark statistically significant decreases in average performance compared to the combined model “−*None*”.

Model performance was measured by calculating the macroaveraged mean absolute error per trial and then averaging over all the testing trials. The average performance of every set of 10 models is shown in Figure 12. The bars labeled “−*None*” display the average performance of the models in which no sensors were held out. The labels for the remaining bars indicate the sensor type that was held out. Error bars display the standard deviation of performance across the 10 models. The Kruskal-Wallis test was used to determine whether the observed differences in performance between the holdout models and the combined models are statistically significant; an asterisk indicates *p* < 0.05. For certain adjectives, some EPs perform better than others. For example, *Fast Slide* outperforms the other EPs for **rough**. Additionally it is clear that certain sensory modalities are important for modeling particular adjectives, and that these influential sensors can differ across EPs for a single adjective.

## 4. Discussion

In this paper, we set out to introduce a new learning method for predicting perceptual distributions of haptic adjectives from single interactions. We used this method to test the effectiveness of certain exploratory procedures and sensory modalities on haptic adjective prediction. The presented results demonstrate that our proposed learning method can successfully model a distribution of possible adjective labels for a single interaction with an object that has never been previously touched. Additionally, we found that certain sensory modalities and exploratory procedures were more significant to predicting specific haptic adjectives than others. The analysis of the human labels allows us to evaluate how people interpret the meaning of certain haptic adjectives and whether the adjective pairs are indeed used as antonyms.

### 4.1. Human Labels

Haptics researchers have proposed the 10 adjectives we studied as possible antonym pairs representing both relevant and primary dimensions of perception. We wanted to further test these propositions and also validate the collected labels for our subsequent machine-learning investigations.

Interestingly, we found that the study participants used some haptic adjective scales more consistently than others. These patterns may stem from underlying dis/agreement about the definitions of the employed adjectives, or they might come from the design of our experiment, such as the chosen set of objects. **Sticky** stands out as having high median agreement with low variation in agreement across objects. As seen in Figure 4, only one object (Silicone Block) was rated “very **sticky**.” Most other objects were rated “not at all **sticky**,” yielding the overall high agreement about the use of this adjective. **Sticky** has no strong positive or negative correlations with the other studied adjectives, but this is because there are very few objects that were rated as sticky. Thus, we cannot make strong claims about the relationship between **sticky** and other haptic adjectives.

The full 1–5 scale was used much more frequently for **hard**, **cold**, **warm**, and **rough**. Thus, we believe their high median agreement and relatively small agreement variation across objects indicates that participants were generally consistent with one another in how they applied these haptic adjectives. Indeed, all four of these adjectives have only one physically relevant definition in a modern American dictionary (Stevenson and Lindberg, 2010), with the possible exception of **warm**, whose physical definitions pertain both to temperature itself and to the ability of a material to keep the body warm. It is thus reasonable to expect that all subjects were applying approximately the same definition as they made their **hard**, **cold**, **warm**, and **rough** ratings. The weak, significant antonym relationship between **cold** and **warm** reinforces the conclusion that subjects used these adjectives consistently; a stronger antonym correlation might have been observed if we tested thermal adjectives that were more closely matched in intensity, such as cool/warm or cold/hot. Interestingly, we did find significant correlations between **hard** and **cold** despite the strong agreement about definitions that don't seem related. This phenomenon could be explained by hedonics, which argues that human sensory perception is affected by emotional attributes. For example, **hard** and **cold** could be correlated with higher arousal, whereas **soft** and **warm** might be correlated with higher comfort (Guest et al., 2010).

Subjects used the full range of ratings for both **soft** and **slippery** but agreed less on their use than on that of the aforementioned adjectives. The disagreement about **soft** most likely stems from the fact that it has two distinct physically relevant meanings (Stevenson and Lindberg, 2010): one pertains to being easy to compress (the antonym to **hard**, as substantiated by a strong negative correlation between these adjectives), while the other pertains to texture. In contrast, **slippery** has only one physical definition (Stevenson and Lindberg, 2010), so the disagreement on its use may instead stem from disagreement about intensity – how **slippery** is “very **slippery**?”

The relatively low agreement about the words **smooth**, **moldable**, and **springy** may be a warning to other researchers interested in using these words in their studies. As with **slippery**, subjects used the full range of ratings for **smooth**; this haptic adjective has only one definition (Stevenson and Lindberg, 2010), so the observed disagreement most likely stems from variations in how people perceive smoothness intensity. We do not know why this adjective's use suffered more than others from the fact that we did not provide adjective definitions or ground our scales with physical examples. Encouragingly, **smooth** was reliably used as an antonym to **rough**, again substantiating our belief that variations in scaling (and not the fundamental definition of the word) are responsible for **smooth**'s low interrater agreement.

In contrast to the other eight adjectives, **moldable** and **springy** are uncommon words in American English; **moldable** does not even have its own dictionary entry (Stevenson and Lindberg, 2010). Thus we believe that a lack of knowledge of the intended meanings of these adjectives (centered on whether the surface quickly returns when pressed and released) prevented subjects from being able to apply them consistently. This physical property is also difficult to judge on hard materials, as they do not deflect perceptibly when squeezed; consequently, the disagreement about **moldable** and **springy** may simply reflect a human inability to perceive such differences for many of the chosen objects. Without guidance, it seems that participants use both of these words in a similar way as **soft**.

These findings validate the collected labels and shed insights on how these 10 haptic adjectives are used by everyday Americans. We believe other researchers studying human and robot perception of haptic properties will be able to design their own studies more efficiently by considering these results.

### 4.2. Model Performance and Influence of Sensory Modalities

The variance of human perception is rarely represented in the labeling of data or captured by machine learning. However, our proposed method demonstrates that it is indeed possible to model this variance. We found interesting differences in performance across adjectives and across EPs within single adjectives. Additionally, by holding out each sensor modality separately and training multiple models with the same architecture, we were able to measure whether certain tactile data types are better predictors of certain adjectives within single exploratory procedures. Many of the results make intuitive sense, suggesting that our method captures relevant structure that can describe various haptic attributes. As far as we are aware, ours is the first method to predict the probability distribution over an ordinal variable from a single test trial.

For discrimination of **hard**, *P*_*DC*_ seems to be the single most important sensor modality; the increase in error for the EP *Squeeze* is by far the largest increase for any holdout model for the adjective **hard**. Surprisingly, *T*_*DC*_ is also a valuable predictor. However, this finding could be explained by the positive correlation between **hard** and **cold**, as shown in Figure 10. Similar patterns are apparent in the perception of **soft**; again, pressure and temperature seem to be important contributors. However, in this case the spatially distributed fingertip deformation readings, *E*_1:19_, are more important than *P*_*DC*_, probably because the perception of **soft** heavily relies on cutaneous information (Srinivasan and LaMotte, 1995).

**Rough** and **smooth** are more texture-related properties than **hard** or **soft**. As might be expected, they depend more on *P*_*AC*_, *P*_*DC*_, and *E*_1:19_. However, overall performance is weak, which could explain why no individual sensor contributes to prediction dramatically more than any other. This low performance aligns with previous analysis of this dataset, which found that it is difficult to accurately predict **rough** and **smooth** even in a simpler binary classification task (Chu et al., 2015), most likely due to the degradation of the BioTac surface ridges over the course of data collection.

For **slippery**, it is interesting that the only large increases in error occur when *T*_*AC*_ is held out, and that these increases occur only for *Slow Slide* and *Fast Slide*. Such behavior is reasonable because **slippery** pertains to sliding friction and has a relatively strong correlation with **cold**. However, it is surprising that the electrodes *E*_1:19_ don't seem to play a significant role. For *Squeeze* and *Hold*, it seems like slip information is encoded in every sensor, although performance is weaker on average. The models predict **sticky** very well. However, this good performance is almost certainly because the labels for **sticky** have a strong bias toward “not at all **sticky**,” which makes it easier to learn a model for **sticky** from these data. As such, it is more accurate to say that the robot learned only an absence of **sticky**, and not actually the feeling of **sticky**.

**Cold** is influenced more by pressure than by temperature sensors, whereas **warm** is influenced more by the temperature modalities. Although it is not surprising that *P*_*AC*_ is so important to prediction for *Fast Slide*, given the dynamic nature of this EP, it is surprising that *P*_*AC*_ seems to have more influence on temperature-related adjectives than texture-related adjectives. This unexpected dependence on pressure could be a limitation of the object set, in that a majority of the thermally conductive objects are both **hard** and **smooth**. It is possible that these correlated properties are easier to detect than **cold** itself. **Warm** depends more on temperature sensors, which is reasonable given that it was found to be more independent from the other adjectives than **cold**.

The models for **moldable** and **springy** depend on many of the same sensor modalities. For both adjectives, the electrodes *E*_1:19_ are significant for every EP. Additionally, the EPs *Squeeze* and *Slow Slide* are both dependent on *T*_*AC*_. These sensor modality influences are similar to those for **soft**. Interestingly, both of these adjectives are highly correlated with **soft** and each other, as shown in Figure 10. This finding may demonstrate that certain object properties that are significant to humans' judgment of multiple haptic attributes are being captured by the robot sensors and used in the modeling of adjectives.

There are a variety of potential limitations to our implementation of these methods. Particularly, the dictionaries were not optimized for this learning task. Thus, it is possible that certain sensory modalities provided less information than might be expected. Additionally, the individual sensor models were optimized separately from the combined model. By optimizing the individual and combined models simultaneously, the learned representations could likely be improved.

We also did not evaluate the model performance as a function of the number of random samples taken from the label distributions. Undersampling could prevent models from learning how the distribution of labels correlates with the tactile data, whereas oversampling could cause the model to overfit the object label distributions. A potentially useful improvement could be to determine how many random samples to take given the total number of ratings for a particular object-adjective pair. Additionally, evaluating whether certain training samples appear to be outliers from the primary response distribution could be useful. Similarly, we did not look deeply into performance on a per-object basis. Our initial analysis demonstrated that some models perform terribly on one or two objects while performing excellently on the majority. Using a larger and more diverse set of objects and collecting ratings from more human subjects would likely improve all of our results.

Because our ordinal regression method evaluates each adjective individually, it ignores the strong positive and negative correlations between adjectives. It might be possible to improve both performance and training efficiency by implementing an algorithm that can learn all adjectives simultaneously, therefore incorporating these inter-adjective relationships into the learning process.

In our results, we analyzed how the models performed over the full range of responses when data from certain sensors were removed. However, it is possible that certain sensory modalities might not have equivalent predictive power across the full response range. For example, to determine the probability distribution of an interaction for the adjective **rough**, a model could use *P*_*DC*_ to make a distinction between the ratings {1,2,3} and {4,5}, but be unable to use it to discern ratings within those two groups. Similarly, the electrodes *E*_1:19_ could provide information that allows the model to discriminate between ratings 4 and 5. Analyzing how the contributions of sensor modalities vary across the full range of ratings could provide greater insight into what type of information is used to determine the haptic attributes of objects.

### 4.3. Conclusion

Machine learning in haptics research often ignores the richness of human perception, instead reducing natural variance to a binary metric. We used the large PHAC-2 dataset to present and analyze labels that represent the range of human haptic attribute perception more granularly than traditional binary labels, also validating the antonym pairs of **hard**–**soft**, **rough**–**smooth**, and **cold**–**warm**. We developed a method that captured this richer information in a model, which could then be used to predict probability distributions of all 10 haptic adjectives for objects that had never been touched before. We believe this research is an important step toward fully capturing the robustness and richness of human haptic perception. Furthermore, because unsupervised dictionary learning and our new method are easily adapted to different sensor and data types, we believe our research broadens the range of tasks that can be tackled with machine learning.

## Author Contributions

BR defined the proposed methodology, performed all machine-learning experiments, analyzed the corresponding results, wrote the majority of the manuscript, and edited the final manuscript. KK designed and supervised the collection of the PHAC-2 dataset, analyzed the interrater agreement and correlations for the adjective labels, wrote the corresponding descriptions and analyses, and edited the final manuscript.

### Conflict of Interest

The authors declare that the research was conducted in the absence of any commercial or financial relationships that could be construed as a potential conflict of interest.

## Supplementary Material

Two videos of the PHAC-2 experiments are included. The first demonstrates the robot exploration experiment, and the second demonstrates the human exploration experiment.
